# Acute toxicological evaluation of AT-533 and AT-533 gel in Sprague-Dawley rats

**DOI:** 10.1186/s40360-023-00696-5

**Published:** 2023-10-13

**Authors:** Lishan Zhong, Yanting Wu, Chen Huang, Kaisheng Liu, Cui-fang Ye, Zhe Ren, Yifei Wang

**Affiliations:** 1 Department of Cell Biology, College of Life Science and Technology, Jinan University, Guangzhou, China; 2 Guangdong Province Key Laboratory of Bioengineering Medicine, Guangzhou, China; 3 Guangdong Provincial biotechnology drug and Engineering Technology Research Center, Guangzhou, China; 4 Guangzhou (Jinan) Biomedical Research and Development Center Co. Ltd, Guangzhou, China; 5 Guangdong Provincial Clinical Research Center for Geriatrics, Shenzhen Clinical Research Center for Geriatrics,Shenzhen People’s Hospital, The Second Clinical Medical College, Jinan University, Shenzhen, Guangdong China; 6 Shenzhen People’s Hospital, The First Affiliated Hospital, Southern University of Science and Technology, Shenzhen, Guangdong China

**Keywords:** AT-533, AT-533 gel, Acute toxicity, Sprague-Dawley rats

## Abstract

**Background:**

AT-533 is a novel heat shock protein 90 inhibitor that exerting anti-inflammatory, antiviral, and antitumor efficacy. Furthermore, the gel made of AT-533 as raw material named AT-533 gel has the function of repairing keratitis and dermatitis caused by herpes virus infection. However, the acute safety evaluation of AT-533 and AT-533 gel has not been conducted.

**Methods and results:**

Herein, we performed acute toxicological studies of AT-533 and AT-533 gel in Sprague-Dawley rats. Fifteen-day acute toxicity study of AT-533 was conducted in both male and female Sprague-Dawley rats at doses of 5, 50, 250 and 500 mg/kg and AT-533 gel at 5 g/kg in the study. During experiment, food consumption and mortality were observed and body weight, hematology, serum biochemistry and histopathological assessment of rats were carried out. No abnormal changes were observed in rats percutaneously treated with AT-533 at 5 mg/kg and 50 mg/kg and AT-533 gel. However, loss of appetite and body weight, adverse reactions, toxicologically relevant alterations in hematology and biochemistry were found in rats percutaneously treated with AT-533 at 250 mg/kg and 500 mg/kg during 15-day acute dermic toxicity study.

**Conclusions:**

The aforementioned results suggested that the LD_50_ of AT-533 is 228.382 mg/kg and the LD_50_ of AT-533 gel is greater than 5 g/kg. These findings indicated that AT-533 is non-toxic in rats when the dose less than 50 mg/kg and AT-533 gel can be considered a gel with no toxicity at doses less than 5 g/kg.

## Introduction

Heat shock protein (HSP) was first discovered by Italian scientist Ferruccio Ritossa in the 1960s [1].Based on their molecular weight, HSPs are classified into five families: HSP110 (HSPH), HSP90 (HSPC), HSP70 (HSSPA), HSP60 (HSPD), and small HSPs (e.g., HSPB1 ) [2]. It is a molecular chaperone of 90 kDa that binds to and activates client proteins in ATP-dependent pathways [3] .Chaperones such as HSP90 assist the conformational maturation, folding, and refolding of client proteins during stress [4] .It regulates multiple critical cellular processes through its interactions with them, including cell proliferation, cell cycle, signaling transduction, and tumor progression. Hsp90 expression is increased in several types of cancer, including breast, ovary and prostate cancers, glioblastoma, melanoma, and hepatocellular carcinomas [5].It is known that cancer cells need HSP90 to maintain their homeostasis, where mutated or overexpressed oncoproteins are stabilized. Therefore, HSP90 is an attractive therapeutic target for cancer treatment.

As HSPs rise, some viruses can take advantage of the activated cellular stress response and increase their replication. The E protein of dengue virus (DENV) interacts with HSP90, resulting in a decrease in cellular E protein and an increase in viral titers [6]. HSP90 is a chaperone of various key regulators related to cell growth and survival [7] and used as a therapeutic target for the treatment of cancer [7–9], virus [10], autoimmune and inflammatory diseases [11].

At least 18 Hsp90 inhibitors have entered clinical trials targeting NTD since the discovery of the first inhibitor, geldanamycin, in 1994 [12]. There are multiple promising Hsp90 inhibitors in clinical trials, including Onalespib [5].

In latest ten years, we have synthesized a series of 2-aminobenzamide derivatives as novel potent Hsp90 inhibitors, including AT-533 (SNX-25a), SNX-7081 and BJ‑B11 [13–15]. All of them are novel analogue of SNX-2112 [16] and has been optimized through structure-activity relationship (SAR) exploration to achieve high Hsp90 affinity [13, 15]. In our previous studies, AT-533 was demonstrated to have many therapeutic potential, including antitumor, antiviral and anti-inflammatory properties [13, 17–19]. For example, AT-533 has broad spectrum and high selectivity in inhibiting tumorigenic properties of various cancer cells [20], and inhibits breast cancer growth through HIF-1α/VEGF/VEGFR-2-mediated angiogenesis. Moreover, AT-533 reduced HSV‑1‑induced the nuclear translocation of NF-κB and NLRP3 inflammasome activation *via* inhibition of the chaperone function ofHSP90 [19]. In addition, AT-533 plays an inhibitory effect on human herpes simplex virus type 1 (HSV‑1) by blocking HSV‑1 nuclear egress and assembly [18] and preventing HSV-1 replication and entry in vitro [21, 22]. Furthermore, AT-533 gel, the gel made of AT-533, has been proved efficiently ameliorating HSV‑1 infected keratitis in a rabbit keratitis model [13] and HSV‑1‑induced skin lesions in C57BL/6 mouse zosteriform [23].

Besides the effectiveness and quality, safety is also an important factor in the development of new drugs [24]. Adverse effects are the major challenge in drug development, and in vivo studies evaluate the toxicity of new therapeutic drugs are critical to support research aimed at evaluating the safety and efficacy of randomized controlled clinical trials in humans. As a compound with varied therapeutic potential, no acute toxicity effect is discussed in AT-533 and AT-533 gel treatment in animal models. In the present study, acute toxicological experiments of AT‑533 and AT‑533 gel were carried out on Sprague-Dawley (SD) rats in accordance with the relevant guiding principles [24], in order to determine the non‑toxic dosage and potential acute toxicity in rats. The present study provides a useful reference for further preclinical research into the use of AT‑533 gel in animals and its clinical application as a skin medication for humans.

## Materials and methods

### Test substances

AT-533 (Lot No. 20180520) and AT-533 gel (Lot No. 20180706) were acquired according to previous reports [25, 26]. AT‑533 was dissolved in propyleneglycol, PEG400 and sterile water for skin absorption. All solvents meet the requirements of the Chinese Pharmacopoeia [27].

### Animals and experimental procedure

All animal experiments were carried out in accordance with the National Institutes of Health’s Guide for the Care and Use of Laboratory Animals. All animal protocols were approved by the Animal Experiment Center of Jinan University. Ninety specific pathogen‑free (SPF) male and female 6-8-week old SD rats were obtained from the Experimental Animal Center of Southern Medical University (Guangzhou, China) under the license number SCXK(YUE)20160041. Feeding conditions are consistent with what we described earlier [26]. Simply, every 5 rats was kept in a plastic cage with labels attached for identification. The rats were housed in an animal room with a 12/12 h light/dark cycle under standard conditions of temperature (21–25℃) and humidity (60–80%) and had free access to food and water. After ten days of acclimation, the fur (about 16 cm^2^) of the rats from the back to the abdomen were removed by pet hair shaver. Then, the depilatory cream was uniformly applied to the area where the hair had been removed to take off the hair thoroughly. After that, the rats was put into the cage for overnight and was recovered from the stimulation of the depilating cream. The next day, for testing AT-533, 60 rats were randomly divided into 6 groups, including the control, vehicle, AT‑533 (5, 50, 250 and 500 mg/kg). The rats in the control group were administered with sterile saline and the rats in the vehicle group were administered with solvents, respectively. Rats in AT-533 group were transdermally given a corresponding dose of the AT-533. For AT-533 gel, 30 rats were randomly assigned to control group, blank gel group, 5 g/kg AT-533 gel group. Rats in corresponding group were transdermally received sterile saline, blank gel and AT-533 gel, respectively. A total of 5 female rats and 5 male rats were assigned to each group. All rats were transdermally treated with solvent or test substance one time. The test substance was applied directly to the skin and exposure was for 24 h. Then, the test substance was wiped off using warm water.

### Observation and detection indicators

All rats were immediately observed after AT-533 or AT-533 gel administration, the animal’s reaction can be observed three times on the day of administration, and then at least once a day for 15 consecutive days of observation period to record any delayed toxic effects. Daily observation records included body weight, skin, fur colour, eyes, autonomic activity, diet, defecation, behaviour of the central nervous system, survival and so on. On day 16 after administration, all rats were anesthetized with pentobarbital sodium (60 mg/kg, i.p.), and blood samples from abdominal aorta were collected for blood routine and biochemical examinations. After that, gross necropsy and anatomy were performed. All the vital organs such as heart, liver, spleen, lung, kidneys and brain were separated and weighed. The organ coefficients (organ-to-final-body-weight ratios) were calculated. Finally, the median lethal dose (LD_50_) (mg/kg) of AT-533 and AT-533 gel was calculated depended on the number of rats that died during the 15-day acute toxicity test.

### Hematological and biochemical parameters

In this study, hematological examination items include white blood cell count (WBC), erythrocyte count (RBC), hemoglobin concentration (HGB), hematocrit (HCT), mean corpuscular volume (MCV), mean corpuscular hemoglobin (MCH), mean corpuscular hemoglobin concentration (MCHC), red cell distribution width (RDW), platelet count (PLT) and mean platelet volume (MPV) norepinephrine (NE), lymphocyte (LY), eosinophil (EO), basophilic granulocytes (BA) and monocytes (MO).

Biochemical indexes include total serum protein (TP), albumin (ALB), aminotransferase (ALT), serum aspartate aminotransferase (AST), total bilirubin (T-Bil), direct bilirubin (D-Bil), indirect bilirubin (I-Bil), alkaline phosphatase (ALP), creatinine (CRE), urea nitrogen (BUN), uric acid (UA), triglycerides (TG), blood glucose (GLU), total cholesterol (CHO), high-density lipoprotein cholesterol (HDL), low density lipoprotein-cholesterol (LDL), sodium (Na), potassium (K), and chloride (Cl).

### Histopathological assessment

The following vital organs were obtained during necropsy: brain, heart, liver, lung, spleen, kidney and skin. Histological examination for the present study was similar to the procedure in our previous study [28]. Briefly, fresh tissue (1.5 × 1.5 × 0.5 cm) was fixed in 4% paraformaldehyde for 8 h at room temperature, dehydrated using an alcohol gradient, and embedded in paraffin. The tissue was then sectioned into 3-µm slices and stained with hematoxylin and eosin (H&E) and assessed for histopathology using a light microscope (Nikon Corporation, Tokyo, Japan). Besides, calculations were made of the organ coefficients (organ-to-final body-weight) before fixation.

### Statistical analysis

All data was analyzed using GraphPad Prism 6.02 (Graph Pad Software, La Jolla, CA, USA) and Microsoft Excel 2013 and presented as mean ± SD. The median lethal dose (LD_50_) (mg/kg) was calculated by Karber’s method [29], whose basic operation formula is as follows: lgLD_50_=∑1/2(Xi + Xi + 1)(P i + 1-Pi). Among them, Xi means dose logarithm and Pi means mortality rate. Statistical significance was determined using one-way ANOVA (and nonparametric) followed by Dunnett’s Multiple Comparison Test. The significant difference was indicated by P values less than 0.05, 0.01, 0.001 or 0.0001.

## Results

### Laboratory observation, food consumption and body weight

**Fig. 1:**
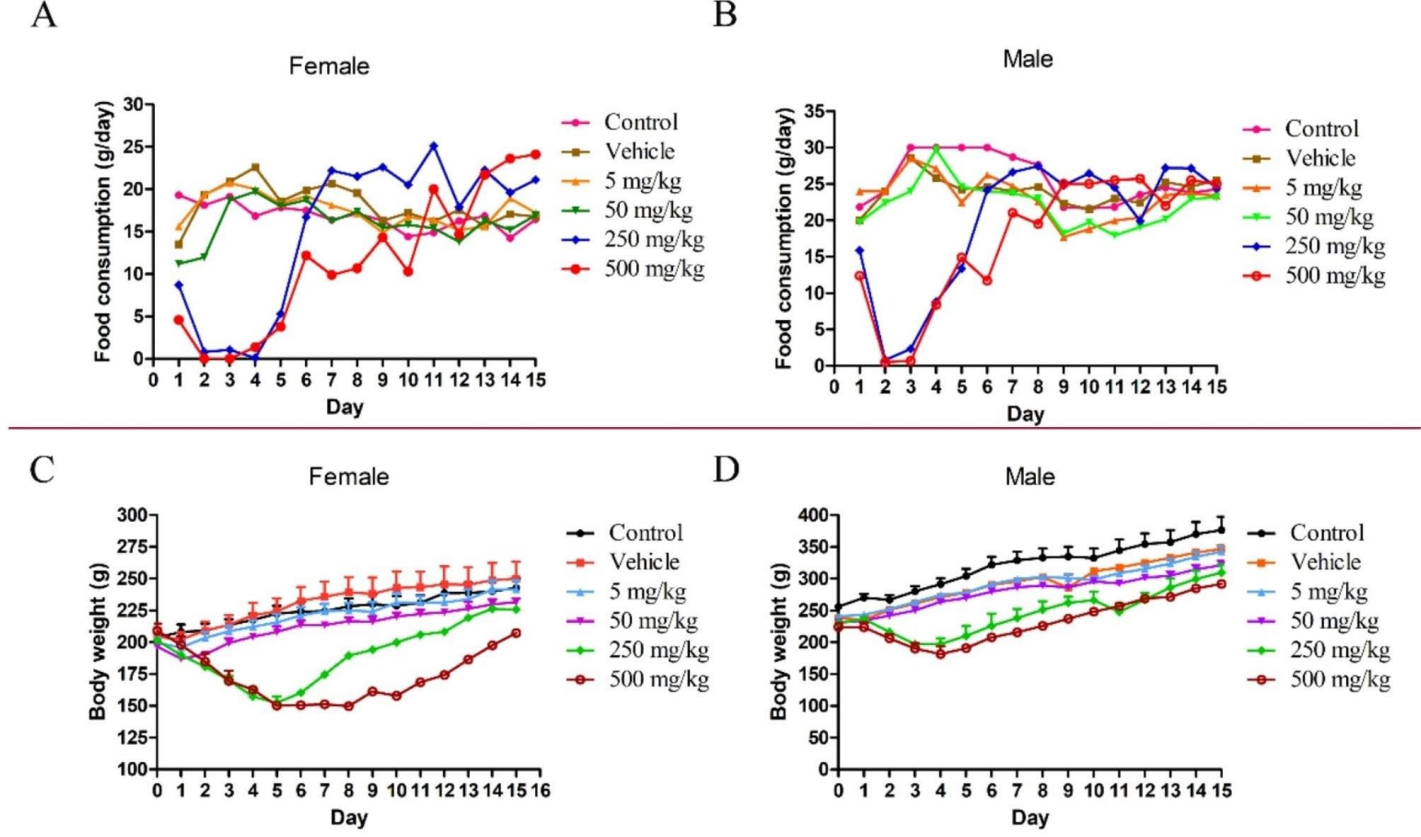
Changes of food consumption and body weight of SD rats of different doses of AT-533. (**A**) Average daily food consumption of female rats. (**B**) Average daily food consumption of male rats. (**C**) Average body weight of female rats. (**D**) Average body weight of male rats. Data presented as mean ± SD (n = 5). A separate one‑way ANOVA analysis was performed for each time point. *P < 0.05, **P < 0.01, ***P < 0.001, ****P < 0.0001 vs. vehicle group

In the acute toxicity test of AT-533, 5 deaths (1 male and 4 females) of 250 mg/kg group and 8 deaths (4 males and 4 females) of 500 mg/kg group were recorded during the study. Accordingly, the LD_50_ of AT-533 calculated using the Karber’s method [29] was 228.382 mg/kg. In addition, rats in control group, vehicle group, 5 mg/kg group and 50 mg/kg group showed a normal diet after transdermal administrating with AT-533. On the contrary, rats in 250 mg/kg group and the 500 mg/kg group showed appetite loss when transdermal treated with AT-533 for 1 to 5 days, and returned to normal on the 6th day (Fig. 1A**&B**). Correspondingly, the body weight of rats showed the same floating trend (Fig. 1C**&D**).

**Fig. 2:**
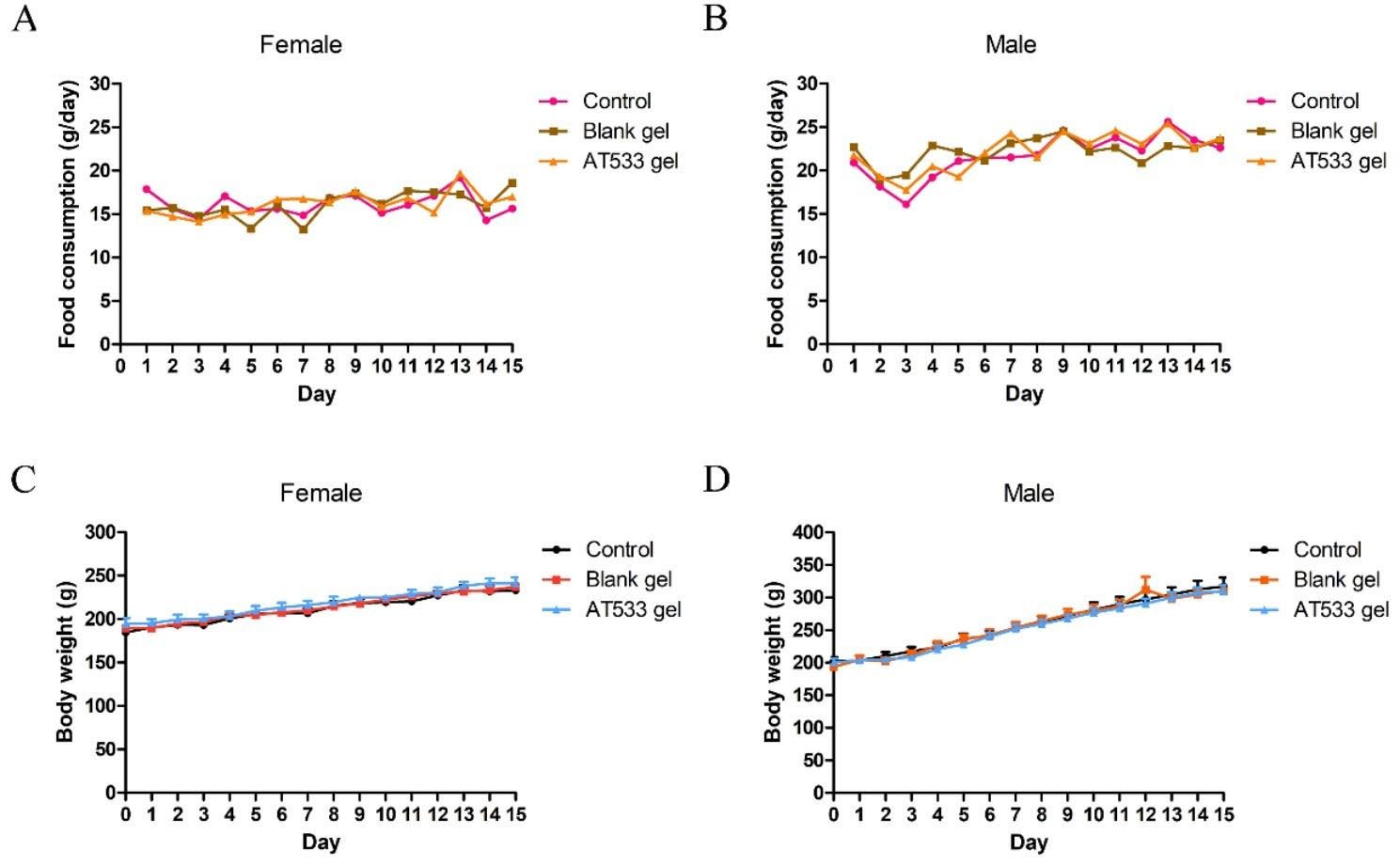
Changes in food consumption and body weight of SD rats after transdermal administration of a single dose of AT-533 gel (5 g/kg). (**A**) Average daily food consumption of female rats. (**B**) Average daily food consumption of male rats. (**C**) Average body weight of female rats. (**D**) Average body weight of male rats. Data presented as mean ± SD (n = 5)

In the acute toxicity test of AT-533 gel, no death was recorded and no abnormal symptom was observed during the study. Thus, LD_50_ of transdermal administration with single dose of AT-533 gel was greater than 5 g/kg. Normal body weight and food consumption were measured in male and female of three groups (Fig. 2).

### Hematology and biochemical examination

When compared with the control group, there was no significant difference in hematology indexes between the vehicle group and the control group. From the results of hematology examinations, the PLT of rats in 500 mg/kg group was remarkably higher than vehicle group rats. In addition, there was no significant difference in hematology index among rats in the vehicle group, 5 mg/kg group, 50 mg/kg group and 250 mg/kg group (Table 1). From the results of biochemical examinations, no significant difference in biochemical items between rats in the vehicle group and the control group was observed. However, ALP for rats of 250 mg/kg group and ALP, CHO, HDL and K^+^ for rats of 500 mg/kg group were remarkably higher than those of vehicle group rats (Table 2).

**Table 1:** Hematological values of rats treated with AT-533

Parameters	Control	Vehicle	5 mg/kg	50 mg/kg	250 mg/kg ^a^	500 mg/kg ^b^
WBC (×10^9^/L)	10.588 ± 2.553	10.187 ± 2.233	9.951 ± 3.668	7.650 ± 2.673	8.680 ± 2.691	9.120 ± 4.327
RBC (×10^12^/L)	8.669 ± 0.875	8.769 ± 0.723	8.472 ± 1.731	9.341 ± 1.025	8.650 ± 0.870	7.860 ± 0.212
HGB (g/L)	189.500 ± 18.429	189.700 ± 14.213	210.700 ± 20.913	191.300 ± 23.109	184.600 ± 18.716	156.000 ± 4.243
HCT (%)	54.610 ± 5.864	55.310 ± 3.844	55.922 ± 7.724	57.130 ± 6.898	55.640 ± 5.664	47.250 ± 3.465
MCV (fl)	63.030 ± 3.447	63.150 ± 2.414	62.460 ± 2.881	61.160 ± 2.800	64.380 ± 2.509	60.100 ± 2.828
MCH (Pg)	21.940 ± 1.947	21.650 ± 0.617	24.322 ± 5.617	20.490 ± 1.135	21.360 ± 1.139	19.850 ± 0.071
MCHC (g/L)	348.000 ± 23.161	342.900 ± 7.767	385.556 ± 89.083	335.100 ± 16.549	331.600 ± 6.189	331.000 ± 15.556
RDW (%)	14.550 ± 1.311	14.690 ± 0.915	17.300 ± 6.748	15.450 ± 1.160	16.740 ± 0.503	16.950 ± 0.071
PLT (×10^9^/L)	540.556 ± 124.175	520.667 ± 201.604	518.667 ± 116.387	523.222 ± 101.206	465.000 ± 129.524	933.500 ± 92.631^*^
MPV (fl)	6.110 ± 0.671	6.160 ± 0.747	6.420 ± 0.464	6.490 ± 0.702	6.700 ± 0778	6.100 ± 0.424
NE (×10^9^/L)	3.051 ± 1.778	3.103 ± 1.985	2.572 ± 1.171	2.379 ± 0.798	2.655 ± 0.665	3.030 ± 1.952
NE% (%)	23.979 ± 8.502	29.119 ± 8.283	25.889 ± 5.137	28.728 ± 8.553	25.906 ± 10.537	31.690 ± 6.392
LY (×10^9^/L)	6.976 ± 1.805	7.497 ± 2.412	6.599 ± 2.541	5.955 ± 1.845^*^	5.886 ± 2.056	5.640 ± 2.192
LY% (%)	66.452 ± 11.824	67.747 ± 9.875	64.331 ± 8.608	65.039 ± 7.785	68.030 ± 11.234	63.265 ± 5.975
EO (×10^9^/L)	0.094 ± 0.094	0.050 ± 0.055	0.111 ± 0.083	0.073 ± 0.070	0.086 ± 0.066	0.035 ± 0.021
EO% (%)	0.656 ± 0.598	0.404 ± 0.325	1.922 ± 2.863	0.842 ± 0.646	0.960 ± 0.622	0.365 ± 0.035
MO (×10^9^/L)	0.421 ± 0.136	0.490 ± 0.125	0.558 ± 0.259	0.393 ± 0.202	0.408 ± 0.243	0.405 ± 0.148
MO% (%)	3.994 ± 0.783	4.537 ± 1.050	6.447 ± 1.589	5.091 ± 1.498	4.686 ± 1.892	4.550 ± 0.552
BA (×10^9^/L)	0.047 ± 0.051	0.015 ± 0.021	0.052 ± 0.044	0.025 ± 0.031	0.040 ± 0.039	0.010 ± 0.014
BA% (%)	0.336 ± 0.343	0.109 ± 0.114	0.375 ± 0.365	0.298 ± 0.325	0.420 ± 0.375	0.125 ± 0.106

Values are mean ± SD for 10 rats in each group. ^a^Data from 5 rats, ^b^ data from 2 rats. *Statistically significant compared to vehicle (p<0.05).

**Table 2:** Serum biochemical values of rats treated with AT-533

Parameters	Control	Vehicle	5 mg/kg	50 mg/kg	250 mg/kg ^a^	500 mg/kg ^b^
TP (g/L)	58.000 ± 3.357	59.800 ± 3.472	62.530 ± 6.772	61.000 ± 12.544	59.500 ± 7.093	60.500 ± 3.125
ALB (g/L)	29.740 ± 2.068	30.070 ± 1.941	31.360 ± 3.037	29.270 ± 2.547	30.720 ± 3.118	29.900 ± 2.076
ALT (U/L)	45.500 ± 5.297	44.900 ± 6.523	50.900 ± 13.420	46.700 ± 10.111	49.600 ± 13.667	58.000 ± 8.973
AST (U/L)	115.800 ± 25.271	112.400 ± 43.958	138.700 ± 45.078	123.200 ± 38.557	140.600 ± 116.223	157.000 ± 57.865
T-Bil (µmol/L)	0.475 ± 0.213	0.360 ± 0.111	0.334 ± 0.199	0.280 ± 0.163	0.224 ± 0.106	0.170 ± 0.132
D-Bil (µmol/L)	0.097 ± 0.070	0.069 ± 0.047	0.053 ± 0.052	0.032 ± 0.033	0.032 ± 0.038	0.020 ± 0.048
I-Bil (µmol/L)	0.378 ± 0.176	0.291 ± 0.103	0.281 ± 0.171	0.248 ± 0.141	0.192 ± 0.095	0.150 ± 0.098
ALP (U/L)	228.100 ± 68.286	227.700 ± 78.370	245.100 ± 62.413	237.800 ± 75.889	329.400 ± 71.835^*^	343.000 ± 78.632^*^
BUN (µmol/L)	5.053 ± 1.422	5.342 ± 0.966	4.671 ± 1.175	4.810 ± 1.341	4.218 ± 1.187	6.370 ± 1.849
CRE (µmol/L)	33.900 ± 10.316	32.940 ± 5.257	29.170 ± 4.454	25.440 ± 5.071	25.880 ± 9.472	19.800 ± 6.832
UA (µmol/L)	74.590 ± 24.449	66.960 ± 27.964	105.640 ± 34.507	113.690 ± 71.735	101.920 ± 44.647	71.800 ± 56.820
GLU (µmol/L)	12.000 ± 2.939	12.279 ± 3.115	10.993 ± 2.663	11.251 ± 3.348	11.880 ± 2.781	9.660 ± 3.071
TG (µmol/L)	1.099 ± 0.628	1.126 ± 0.563	1.540 ± 0.639	1.527 ± 0.508	1.798 ± 0.570	1.730 ± 0.548
CHO (µmol/L)	1.711 ± 0.186	1.726 ± 0.094	1.717 ± 0.205	1.536 ± 0.186	1.798 ± 0.264	2.140 ± 0.234^*^
HDL (µmol/L)	0.592 ± 0.104	0.627 ± 0.062	0.635 ± 0.092	0.575 ± 0.077	0.716 ± 0.113^*^	0.900 ± 0.124^**^
LDL (µmol/L)	0.201 ± 0.056	0.190 ± 0.045	0.203 ± 0.038	0.186 ± 0.028	0.210 ± 0.025	0.240 ± 0.051
K^+^ (µmol/L)	5.145 ± 0.814	5.000 ± 0.804	5.742 ± 0.623	5.344 ± 0.924	5.842 ± 0.830	6.790 ± 0.649^*^
Na^+^ (µmol/L)	142.640 ± 5.994	143.590 ± 4.939	143.040 ± 6.563	139.590 ± 8.788	144.320 ± 3.648	147.100 ± 6.320
Cl^−^ (µmol/L)	109.970 ± 4.028	111.400 ± 3.722	110.410 ± 4.456	108.420 ± 6.696	111.340 ± 1.278	111.900 ± 3.451

Values are mean ± SD for 10 rats in each group, ^a^ Data from 5 rats, ^b^ data from 2 rats. * Statistically significant compared to vehicle (p < 0.05), ** Statistically significant compared to vehicle (p < 0.01)

In the acute toxicity test of AT-533 gel, the hematology and biochemical results showed no significant differences among control group, blank gel group, AT-533 gel (5 g/kg) group (Tables 3 and 4).

**Table 3:** Hematological values of rats treated with single dose of AT-533 gel (5 g/kg)

Parameters	Control	Blank gel	AT-533 gel
WBC (×10^9^/L)	6.160 ± 1.500	5.931 ± 0.750	7.408 ± 1.684
RBC (×10^12^/L)	8.354 ± 0.827	7.528 ± 1.430	7.533 ± 0.428
HGB (g/L)	191.800 ± 25.793	164.889 ± 22.713	154.000 ± 10.149
HCT (%)	52.630 ± 6.193	46.420 ± 8.394	47.133 ± 2.687
MCV (fl.)	62.920 ± 2.347	61.770 ± 2.601	62.578 ± 1.150
MCH (Pg)	22.920 ± 1.202	20.950 ± 1.216	20.456 ± 0.637
MCHC (g/L)	364.000 ± 10.914	339.000 ± 13.556	326.778 ± 8.318
RDW (%)	14.930 ± 0.750	14.380 ± 1.181	14.689 ± 0.810
PLT (×10^9^/L)	512.400 ± 161.892	682.000 ± 108.510	702.000 ± 126.206
MPV (f.)	6.030 ± 0.855	6.150 ± 1.086	6.544 ± 1.099
NE (×10^9^/L)	2.229 ± 1.201	1.073 ± 0.526	1.951 ± 0.746
NE% (%)	28.861 ± 7.637	21.924 ± 6.882	26.866 ± 6.016
LY (×10^9^/L)	3.934 ± 1.161	3.610 ± 1.359	5.219 ± 1.153
LY% (%)	64.729 ± 6.967	72.214 ± 9.844	68.831 ± 9.050
EO (×10^9^/L)	0.139 ± 0.301	0.096 ± 0.234	0.069 ± 0.100
EO% (%)	0.697 ± 0.460	0.936 ± 0.816	1.368 ± 1.989
MO (×10^9^/L)	0.442 ± 0.334	0.143 ± 0.078	0.246 ± 0.311
MO% (%)	4.890 ± 1.118	3.042 ± 1.436	2.308 ± 1.112
BA (×10^9^/L)	0.070 ± 0.139	0.040 ± 0.109	0.097 ± 0.203
BA% (%)	0.513 ± 0.551	0.256 ± 0.372	0.627 ± 0.951

Values are mean ± SD for 10 rats in each group

**Table 4:** Serum biochemical values of rats treated with AT-533 single dose of AT-533 gel (5 g/kg)

Parameters	Control	Blank gel	AT-533 gel
TP (g/L)	65.110 ± 3.629	62.840 ± 3.208	65.360 ± 3.570
ALB (g/L)	32.040 ± 1.247	30.510 ± 1.639	31.450 ± 2.169
ALT (U/L)	73.900 ± 23.718	72.111 ± 19.154	62.000 ± 15.452
AST (U/L)	106.900 ± 15.638	105.600 ± 21.418	112.400 ± 24.218
T-Bil (µmol/L)	0.638 ± 0.218	0.565 ± 0.270	0.421 ± 0.145
D-Bil (µmol/L)	0.105 ± 0.076	0.097 ± 0.083	0.057 ± 0.028
I-Bil (µmol/L)	0.533 ± 0.215	0.468 ± 0.213	0.364 ± 0.130
ALP (U/L)	180.400 ± 77.090	175.200 ± 79.790	182.000 ± 91.160
BUN (µmol/L)	6.591 ± 1.385	5.898 ± 0.934	7.420 ± 1.456
CRE (µmol/L)	37.220 ± 11.925	35.470 ± 8.359	40.410 ± 11.635
UA (µmol/L)	57.613 ± 15.744	73.770 ± 31.754	82.540 ± 29.092
GLU (µmol/L)	5.557 ± 1.327	7.890 ± 1.306	6.525 ± 1.395
TG (µmol/L)	0.618 ± 0.319	0.630 ± 0.345	0.487 ± 0.188
CHO (µmol/L)	1.586 ± 0.273	1.507 ± 0.258	1.333 ± 0.157
HDL (µmol/L)	0.402 ± 0.132	0.366 ± 0.053	0.334 ± 0.045
LDL (µmol/L)	0.124 ± 0.026	0.115 ± 0.027	0.099 ± 0.017
K^+^ (µmol/L)	4.717 ± 0.813	4.601 ± 1.058	4.613 ± 0.914
Na^+^ (µmol/L)	144.760 ± 1.279	147.770 ± 9.487	148.380 ± 6.093
Cl^−^ (µmol/L)	111.940 ± 2.641	115.930 ± 7.853	115.200 ± 4.934

Values are mean ± SD for 10 rats in each group, ^a^ Data from 5 rats, ^b^ data from 2 rats. * Statistically significant compared to vehicle (p < 0.05)

### Absolute and relative weight of organs

Furthermore, the absolute organ weight and organ coefficients in female and male rats were shown in Tables 5 and 6, which showed no significant difference between the experimental groups (5 mg/kg and 50 mg/kg) and the vehicle group. Since only one female rat survived in 250 mg/kg group and 500 mg/kg group, this part of data was for reference only and was not discussed anymore.

**Table 5:** Absolute and relative weights of organs of female rats treated with AT-533

Parameters	Control	Vehicle	5 mg/kg	50 mg/kg	250 mg/kg ^a^	500 mg/kg ^a^
Body weight (g)	242.82 ± 16.84	250.08 ± 29.75	241.5 ± 16.07	231.62 ± 6.51	225.7 ± 0.01	207.2 ± 0.001^*^
Brain	1.914 ± 0.082	1.871 ± 0.122	1.840 ± 0.073	1.860 ± 0.049	1.610 ± 0.001^**^	1.810 ± 0.001
Heart	0.917 ± 0.048	0.949 ± 0.151	0.938 ± 0.063	0.713 ± 0.399	0.990 ± 0.001	0.810 ± 0.001
Liver	8.196 ± 0.599	8.636 ± 1.245	8.668 ± 0.806	8.434 ± 0.601	9.580 ± 0.001	8.750 ± 0.001
Spleen	0.505 ± 0.072	0.568 ± 0.113	0.652 ± 0.113	0.548 ± 0.043	0.600 ± 0.001	0.500 ± 0.001
Lung	1.266 ± 0.132	1.249 ± 0.133	1.136 ± 0.104	1.266 ± 0.177	1.240 ± 0.001	0.990 ± 0.001
Kidneys	1.704 ± 0.159	1.507 ± 0.230	1.604 ± 0.260	1.622 ± 0.107	2.070 ± 0.001^**^	1.730 ± 0.001
Organ-to-body weight ratio (%)						
Brain	0.789 ± 0.020	0.753 ± 0.051	0.764 ± 0.040	0.803 ± 0.021	0.713 ± 0.001	0.874 ± 0.001^**^
Heart	0.378 ± 0.008	0.378 ± 0.026	0.389 ± 0.019	0.307 ± 0.171	0.439 ± 0.001	0.391 ± 0.001
Liver	3.377 ± 0.157	3.449 ± 0.174	3.590 ± 0.252	3.646 ± 0.327	4.245 ± 0.001^**^	4.223 ± 0.001^**^
Spleen	0.208 ± 0.024	0.227 ± 0.036	0.269 ± 0.037	0.237 ± 0.020	0.266 ± 0.001	0.241 ± 0.001
Lung	0.522 ± 0.054	0.501 ± 0.039	0.470 ± 0.024	0.549 ± 0.094	0.549 ± 0.001	0.478 ± 0.001
Kidneys	0.701 ± 0.029	0.602 ± 0.039	0.663 ± 0.092	0.700 ± 0.035	0.917 ± 0.001^***^	0.835 ± 0.001^***^

Values are mean ± SD for 5 rats in each group, ^a^ Data from 1 rat. * Statistically significant compared to vehicle (p < 0.05), ** Statistically significant compared to vehicle (p < 0.01), ***Statistically significant compared to vehicle (p < 0.001)

**Table 6:** Absolute and relative weights of organs of male rats treated with AT-533

Parameters	Control	Vehicle	5 mg/kg	50 mg/kg	250 mg/kg ^a^	500 mg/kg ^b^
Body weight (g)	376.660 ± 46.935	346.760 ± 14.186	342.340 ± 19.132	321.180 ± 15.806	309.425 ± 26.230	291.800 ± 0.001
Brain	2.005 ± 0.065	1.950 ± 0.116	1.966 ± 0.041	1.984 ± 0.063	1.943 ± 0.052	2.042 ± 0.001
Heart	1.358 ± 0.153	1.251 ± 0.095	1.170 ± 0.104	1.096 ± 0.024	1.165 ± 0.159	1.107 ± 0.001
Liver	12.047 ± 2.594	12.364 ± 0.752	11.922 ± 1.012	11.408 ± 0.867	11.340 ± 0.804	11.816 ± 0.001
Spleen	0.801 ± 0.127	0.764 ± 0.064	0.757 ± 0.210	0.636 ± 0.048	0.719 ± 0.144	0.749 ± 0.001
Lung	1.640 ± 0.137	1.362 ± 0.113	1.577 ± 0.162	1.398 ± 0.196	1.453 ± 0.140	1.836 ± 0.001^**^
Kidneys	2.018 ± 0.530	2.316 ± 0.213	2.053 ± 0.120	2.004 ± 0.216	2.161 ± 0.231	1.824 ± 0.001
Organ-to-body weight ratio (%)						
Brain	0.538 ± 0.063	0.563 ± 0.030	0.575 ± 0.030	0.619 ± 0.039	0.631 ± 0.055	0.700 ± 0.001^**^
Heart	0.362 ± 0.036	0.361 ± 0.030	0.342 ± 0.023	0.342 ± 0.018	0.376 ± 0.032	0.379 ± 0.001
Liver	3.184 ± 0.453	3.565 ± 0.138	3.481 ± 0.201	3.550 ± 0.168	3.673 ± 0.231	4.049 ± 0.001
Spleen	0.215 ± 0.042	0.220 ± 0.014	0.221 ± 0.059	0.198 ± 0.014	0.231 ± 0.034	0.257 ± 0.001
Lung	0.438 ± 0.035	0.394 ± 0.045	0.461 ± 0.050	0.435 ± 0.053	0.471 ± 0.041	0.629 ± 0.001^***^
Kidneys	0.533 ± 0.115	0.667 ± 0.046	0.600 ± 0.026	0.623 ± 0.041	0.698 ± 0.039	0.625 ± 0.01

Values are mean ± SD for 5 rats in each group, ^a^ Data from 4 rats, ^b^ Data from 1 rat. * Statistically significant compared to vehicle (p < 0.05), ** Statistically significant compared to vehicle (p < 0.01), ***Statistically significant compared to vehicle (p < 0.001)

In the acute toxicity test of AT-533 gel, there was no significant difference in organ weight and organ coefficients in female and male rats (Tables 7 and 8). These results suggested that 5 g/kg AT-533 gel was non-toxic to SD rats.

**Table 7:** Absolute and relative weights of organs of female rats treated with AT-533 gel

Parameters	Control	Blank gel	AT-533 gel
Body weight (g)	231.400 ± 13.683	230.960 ± 11.588	233.200 ± 12.787
Brain	1.931 ± 0.065	1.937 ± 0.061	1.865 ± 0.088
Heart	0.980 ± 0.124	0.902 ± 0.058	0.856 ± 0.089
Liver	7.318 ± 1.267	6.619 ± 0.705	6.402 ± 0.367
Spleen	0.639 ± 0.090	0.687 ± 0.148	0.678 ± 0.105
Lung	1.267 ± 0.165	1.229 ± 0.125	1.173 ± 0.098
Kidneys	1.636 ± 0.211	1.660 ± 0.079	1.560 ± 0.057
Organ-to-body weight ratio (%)			
Brain	0.835 ± 0.022	0.840 ± 0.046	0.801 ± 0.047
Heart	0.423 ± 0.044	0.392 ± 0.038	0.367 ± 0.026
Liver	3.153 ± 0.428	2.863 ± 0.220	2.750 ± 0.185
Spleen	0.276 ± 0.036	0.297 ± 0.056	0.292 ± 0.051
Lung	0.548 ± 0.067	0.534 ± 0.069	0.505 ± 0.057
Kidneys	0.705 ± 0.062	0.719 ± 0.025	0.670 ± 0.033

Values are mean ± SD for 5 rats in each group

**Table 8:** Absolute and relative weights of organs of male rats treated with AT-533 gel

Parameters	Control	Blank gel	AT-533 gel
Body weight (g)	298.940 ± 27.022	301.760 ± 13.803	290.060 ± 19.059
Brain	1.974 ± 0.079	1.914 ± 0.065	1.754 ± 0.191
Heart	1.217 ± 0.092	1.283 ± 0.125	1.172 ± 0.149
Liver	8.600 ± 1.072	8.400 ± 0.516	8.036 ± 0.906
Spleen	0.678 ± 0.120	0.641 ± 0.164	0.576 ± 0.087
Lung	1.387 ± 0.079	1.363 ± 0.083	1.344 ± 0.134
Kidneys	2.309 ± 0.155	2.200 ± 0.188	2.114 ± 0.210
Organ-to-body weight ratio (%)			
Brain	0.664 ± 0.058	0.636 ± 0.046	0.610 ± 0.103
Heart	0.408 ± 0.016	0.427 ± 0.057	0.403 ± 0.033
Liver	2.874 ± 0.212	2.785 ± 0.150	2.764 ± 0.150
Spleen	0.226 ± 0.025	0.211 ± 0.045	0.198 ± 0.020
Lung	0.467 ± 0.049	0.452 ± 0.018	0.465 ± 0.054
Kidneys	0.776 ± 0.062	0.729 ± 0.052	0.729 ± 0.053

Values are mean ± SD for 5 rats in each group

### Histopathological examination

**Fig. 3:**
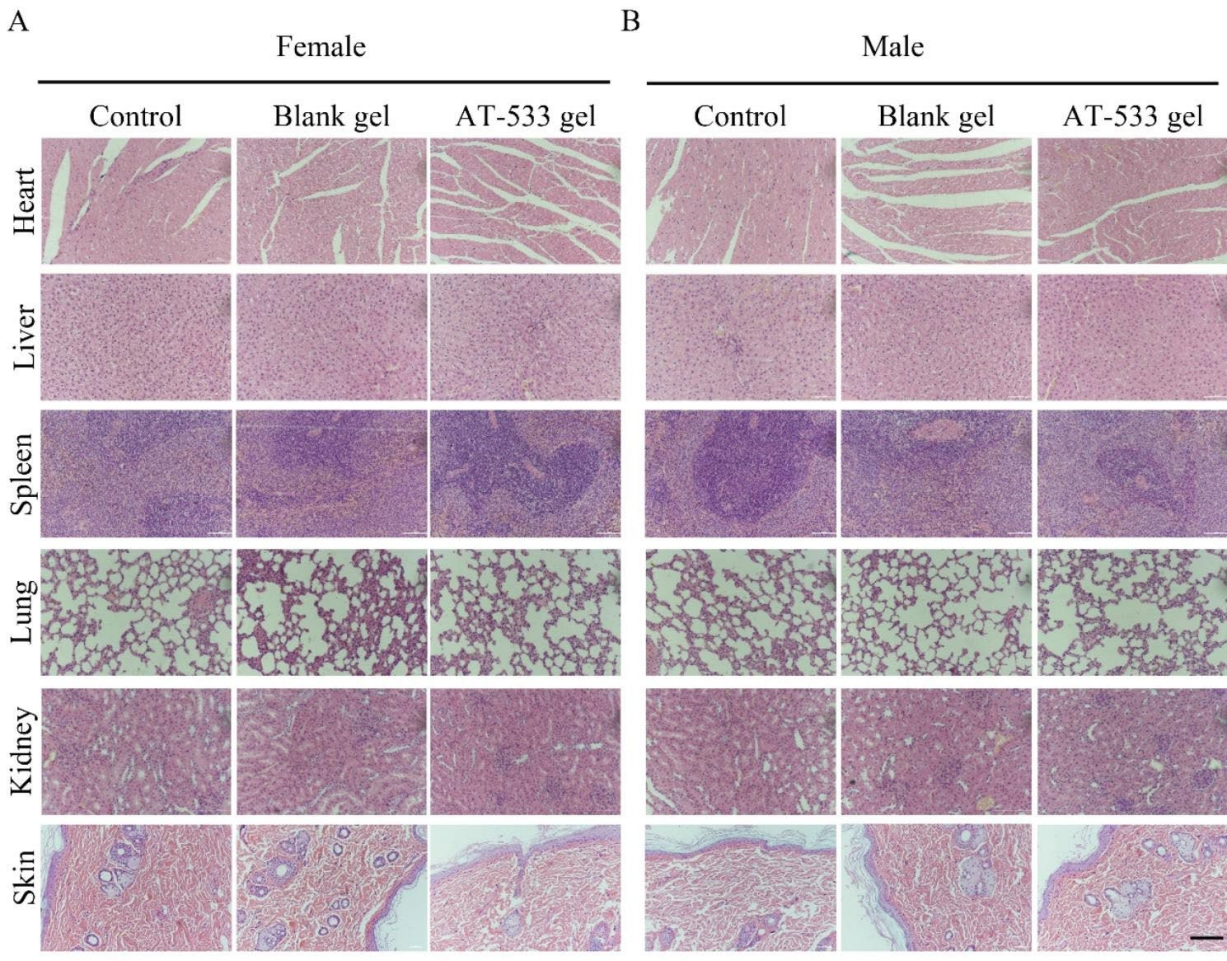
Histological analysis of heart, liver, spleen, lung, kidneys and skin (dosing site) sections from rats treated with 5 g/kg AT‑533 gel in the 15‑day acute toxicity (H&E staining, x 10). Representative micrographs from (**A**) female and (**B**) male rats under the conditions of control, blank gel and AT‑533 gel

Histopathological observations were conducted for organs and tissues of all 15-day toxicity portion rats. There were no pathological lesions on heart, liver, spleen, lung, kidney and shin (dosing site) of female and male rats of 5 g/kg AT-533 gel (Fig. 3). Therefore, these results suggested that AT-533 gel used in this test have no damage effect on internal organs.

## Discussion

As previously pointed out, AT-533 is first synthesized in our laboratory and in the last few years, there has been an expansion of pharmacological studies carried out with this compound, but there still few have addressed toxicological parameters in animal models. As an important part of the development of new drugs, it’s necessary to preform drug safety evaluation. Thus, this study provided the results of 15-day acute toxicity of AT-533 and AT-533 gel in SD rats.

Body weight changes to some extent indicate adverse reactions of drugs [30–32]. In the acute study of AT-533, rats of 5 and 50 mg/kg treated group had normal diet and body weight. However, the food intake, water consumption or weight of rats in the 250 mg/kg and 500 mg/kg treatment group were affected. On the third day of observation, a total of three female rats died at the high doses (250 and 500 mg/kg), indicating that the high doses (250 and 500 mg/kg) AT-533 were toxic and mainly affecting females. The main symptoms of these rats were loss of appetite and diarrhea. No obvious abnormalities were found in other organs except for flatulence, which might be caused by the adverse reaction of AT-533 in stomach. The possible reason maybe the rats licked each other after transdermal administration with AT-533 that cause some drugs reaching stomach through the mouth. From the result, the LD_50_ of AT-533 calculated is 228.382 mg/kg. Our previous in vitro and in vivo studies showed that the effective concentration was much lower than the LD_50_ of AT-533 [18, 19, 23], which confirmed the potential druggability of AT-533. Besides, no death, abnormal diet and body weight were found in rats transdermally treated with 5 g/kg AT‑533 gel.

As hematological parameters are key indicators of physiological and pathological status [33], the blood samples were collected to detected to indicate evaluate the toxic effects of the AT-533 and AT-533 gel. Although there was a significant decreased level of lymphocytes (LY) in rats of 50 mg/kg AT-533 treatment group, it was not considered toxic-related since the value of them stayed within the normal reference laboratory range [34]. The increased PLT in rats of 500 mg/kg AT-533 treatment group maybe result from the side effect of AT-533, but the result from only 2 rats, which may be caused by individual differences. Taking into account individual differences, since only 1 ~ 2 rats survived in AT-533 of 250 mg/kg group and 500 mg/kg group, the biochemical parameters and organ coefficients data were for reference only and were not discussed anymore.

In addition, all acquired hematological parameters of rats treated with 5 g/kg AT-533 gel did not show significant variation among rats of control and blank gel groups, which indicated the possible absence of hematological toxicity. The liver and kidneys are target organs to eliminate of xenobiotics, so their function tests are indispensable. The biochemical examinations of rats treated with 5 mg/kg, 50 mg/kg AT-533 or 5 g/kg AT-533 gel reveal no hepatotoxicity and renal toxicity. Organ weight and organ coefficients are important indicators for evaluating the toxic effects of test substances in preclinical toxicology studies of new drugs, and have a high reference value for target organ confirming drug toxicity [24]. The results of Tables 5, 6, 7 and 8 showed no statistical difference among the groups. Furthermore, both gross and histopathological findings showed normal heart, liver, spleen, lung, kidneys and skin morphology of rats treated with 5 g/kg AT-533 gel.

In summary, when SD rats were treated with AT-533 at doses of 5 mg/kg and 50 mg/kg and AT-533 gel at a dose of 5 g/kg, it did not cause mortality. Additionally, the blood routine, biochemical parameters and organ physiological conditions were normal, which indicates that the safe and worthy of AT-533 and its gel to further promote and develop. Therefore, the LD_50_ of AT-533 is 228.382 mg/kg and the single dose dermic LD_50_ of AT-533 gel for rats is in excess of 5 g/kg. The aforementioned results provide a reference for subsequent subchronic and chronic toxicity tests of AT‑533 and AT‑533 gel.

## Data Availability

All the necessary data used to support the results of this study are included in the manuscript.

## Abbreviations

ALB: Albumin
ALP: Alkaline phosphatase
ALT: Aminotransferase
AST: Serum aspartate aminotransferase
BA: Basophilic granulocytes
BUN: Urea nitrogen
CHO: Total cholesterol
Cl: Chloride
CRE: Creatinine
D-Bil: Direct bilirubin
EO: Eosinophil
GLU: Blood glucose
HCT: Hematocrit
HDL: High-density lipoprotein cholesterol
HGB: Hemoglobin concentration
Hsp90: Heat shock protein 90
HSV‑1: Human herpes simplex virus type 1
I-Bil: Indirect bilirubin
K: Potassium
LD_50_: The median lethal dose
LDL: Low density lipoprotein-cholesterol
LY: Lymphocyte
MCH: Mean corpuscular hemoglobin
MCHC: Mean corpuscular hemoglobin concentration
MCV: Mean corpuscular volume
MO: Monocytes
MPV: Mean platelet volume
Na: Sodium
NE: Norepinephrine
PLT: Platelet count
RBC: Erythrocyte count
RDW: Red cell distribution width
SD: Sprague-Dawley
T-Bil: Total bilirubin
TG: Triglycerides
TP: Biochemical indexes include total serum protein
UA: Uric acid
WBC: White blood cell count
